# Dietary protein score and carbohydrate quality index with the risk of chronic kidney disease: Findings from a prospective cohort study

**DOI:** 10.3389/fnut.2022.1003545

**Published:** 2022-11-30

**Authors:** Farshad Teymoori, Hossein Farhadnejad, Mitra Kazemi Jahromi, Mohammadreza Vafa, Hamid Ahmadirad, Parvin Mirmiran, Fereidoun Azizi

**Affiliations:** ^1^Nutrition and Endocrine Research Center, Research Institute for Endocrine Sciences, Shahid Beheshti University of Medical Sciences, Tehran, Iran; ^2^Department of Nutrition, School of Public Health, Iran University of Medical Sciences, Tehran, Iran; ^3^Endocrinology and Metabolism Research Center, Hormozgan University of Medical Sciences, Bandar Abbas, Iran; ^4^Endocrine Research Center, Research Institute for Endocrine Sciences, Shahid Beheshti University of Medical Sciences, Tehran, Iran

**Keywords:** chronic kidney disease, macronutrients, protein score, carbohydrate quality, adults

## Abstract

**Background/Aim:**

This study aimed to examine the associations between dietary protein score and carbohydrate quality index (CQI) and the risk of chronic kidney disease (CKD) in Iranian adults.

**Methods:**

This population-based cohort study was performed within the Tehran Lipid and Glucose Study framework on 6,044 subjects aged ≥18 years old, who were followed up for a mean of 7.7 years. Dietary protein score and CQI were determined using a food frequency questionnaire. CKD was defined as an estimated glomerular filtration rate <60 ml/min/1.73 m^2^. A multivariate Cox proportional hazard regression model was used to estimate the risk of CKD across tertiles of protein score and CQI.

**Results:**

The mean (standard deviation) of age and body mass index of participants were 37.9 (12.8) years and 26.8 (4.7) kg/m^2^, respectively. During the 7.7 ± 2.7 years of follow-up, 1,216 cases (20.1%) of CKD were ascertained. In the final adjusted model, individuals in the highest tertile of protein score had decreased risk of CKD (HR: 0.85, 95% CI: 0.74–0.98, *P*_trend_ = 0.033). Also, there is a significant association between total carbohydrate score (HR: 0.85, 95% CI: 0.73–0.99, *P*_trend_ = 0.016), the ratio of whole grain/total grains (HR: 0.81, 95% CI: 0.70–0.94, *P*_trend_ = 0.004), and glycemic index (HR: 1.30, 95% CI: 1.12–1.51, *P*_trend_ < 0.001) and risk of CKD. However, no significant association was found between total protein intakes, plant-to-animal ratio, and solid carbohydrate/total carbohydrate with the risk of CKD.

**Conclusion:**

Our results revealed a diet with a high protein score and high quality of carbohydrates, characterized by higher intakes of plant proteins, low glycaemic index (GI) carbohydrates, whole grain, fibers, and lower intakes of animal proteins, can be related to reduced CKD risk.

## Introduction

Chronic kidney disease (CKD) is a progressive disease characterized by the existence of kidney injuries or a glomerular filtration rate (GFR) lower than 60 ml/min/1.73 m^2^ (1). The global prevalence of CKD has been increasing since 1990 and reached 9/1%, equal to 697⋅5 million cases and 1.2 million death in 2017. CKD resulted in 35.8 million disability-adjusted life-years (DALYs) and 1.4 million cardiovascular diseases (CVDs) related deaths in 2017 (2, 3). Diabetes, hypertension (HTN), and lifestyle factors such as obesity, smoking, and bad diet quality have been recognized as risk factors for CKD (4, 5).

Diet as a determinant modifiable related factor with CKD, assessed in various aspects, including dietary macro and micronutrients, food groups, and dietary patterns to predict the risk of CKD in previous studies (6, 7). Carbohydrates and protein, as the main sources of the human diet, form 60 to 75% of a usual diet (8), so they have an important role in body metabolism and human health. Studies showed that the metabolic effects of carbohydrates and protein are influenced by their components and types based on structural features, physiologic behaviors, food sources, and consumption amounts (3, 9).

Findings of a meta-analysis proposed that a high protein compared to a low or normal protein diet increased the GFR in subjects who were free of CKD (10). Also, some studies assessed the sources of protein intake from animal or plant foods with CKD and reported controversial findings (3, 11–13). Recently a protein score that combined total protein and plant-to-animal ratio (PAR) was proposed to predict the quality and quantity of dietary protein intake (12). Møller et al. indicated that higher protein scores are inversely related to HbA1c and positively increased GFR (12).

Carbohydrates are a heterogeneous class of nutrients, and their various types may affect body metabolism differently (14). Fiber is a subclass of carbohydrates that has various beneficial effects, however, simple sugars are easily metabolized into fatty acids and could adversely affect health markers. Also, frequent consumption of food with high glycaemic index (GI) increases the risk of obesity, diabetes, and CVD (15). The results of some observational studies show that the number of carbohydrates in the diet can be important in predicting kidney damage and CKD risk. Yuzbashian et al. reported that there is no significant association between the consumption of dietary carbohydrates and CKD risk (11); however, the Korean Genome and Epidemiology Study showed that a diet with a low fat-to-carbohydrate intake ratio was related to a higher risk of CKD in the general population (16). Furthermore, Farhadnejad et al. suggested that a low-carbohydrate, high-protein diet may be associated with increased CKD risk among Tehranian adults (17). It seems that carbohydrate quality has the same or more important role than carbohydrate amount in human health (18). Therefore nutritionists always recommend improving the quality of carbohydrate intake to prevent diseases (19). Zazpe et al. proposed the carbohydrate quality index (CQI) using four criteria: dietary fiber intake, GI, whole grains/total grains ratio, and solid carbohydrate/total carbohydrate (SCHO/TCHO), with the hypothesis that higher CQI is associated with higher micronutrient intake adequacy and lower risk of chronic disease CQI (20). They found that a higher CQI was strongly associated with better micronutrient intake adequacy (20) and showed a significant inverse association with the incidence of CVD (14). Also, Kim et al. have indicated that a higher CQI is related to a lower risk of obesity and HTN, however, it was not associated with diabetes and metabolic syndrome (MetS) (21).

To our knowledge, the association between protein score and CQI was not previously assessed with the risk of CKD incident, so we aimed to assess the associations between dietary protein score and CQI and the risk of CKD in Iranian adults.

## Materials and methods

### Study population

The current study was performed in the framework of the Tehran Lipid and Glucose Study (TLGS), a population-based cohort study conducted to investigate the risk factors of chronic diseases among a representative urban population of Tehran, including 15,005 participants aged≥3 years (22). The first survey of TLGS was initiated in March 1999, and data collection conducted prospectively at 3 years intervals is ongoing. The baseline survey was a cross-sectional study conducted from 1999 to 2001, and surveys II (2002–2005), III (2006–2008), IV (2009–2011), V (2012–2015), and VI (2015–2018) were prospective follow-up surveys. The details of the TLGS have been explained previously (22).

**FIGURE 1:**
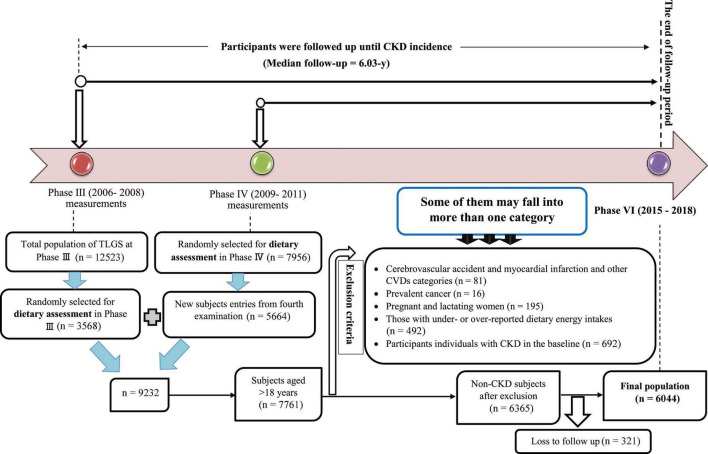
Flowchart of the study population.

In the third survey of the TLGS (2006–2008), of 12,523 participants, 3,568 were randomly selected for dietary assessment, and in the fourth survey (2009–2011), 7,956 randomly selected subjects agreed to complete dietary assessment. For the current study, participants with complete dietary data on the third examination of TLGS and the new entries participants in the fourth examination, which was 9,232, were included. For the present study, of 7,761 individuals aged ≥18 years, participants with CVD outcomes (*n* = 81), prevalent cancer (*n* = 16), pregnant and lactating women (*n* = 195), those with under- or over-reported dietary energy intakes (out of the range 800–4,200 kcal/day) (23) (*n* = 492) and participants with CKD in the baseline (*n* = 692) were excluded. Some of them may fall into more than one category (*n* = 77). Of 6,365 participants at baseline, who were followed-up, 321 were lost to follow-up, and 6,044 participants remained for final analysis (follow-up rate: 94.95%) (Figure 1).

### Ethical statement

The study complies with the Declaration of Helsinki, and the study protocol was approved by the Ethics Committee of the Research Institute for Endocrine Sciences, affiliated with the Shahid Beheshti University of Medical Sciences, Tehran, Iran. Informed consent was obtained from all individual participants included in the study.

### Demographic and anthropometric assessments

Demographic information, including age, sex, smoking status, educational level, medical history, medication use, etc., was assessed using a pretested questionnaire. Weight was measured over light clothing using a SECA digital weighing scale (Seca 707; Seca Corporation, Hanover, Maryland) with an accuracy of up to 100 g. Height was measured using a stadiometer with a minimum of 1 mm in a standing position without shoes and shoulders in normal alignment. Body mass index (BMI) was computed as weight (kg) divided by the square of height (m^2^).

### Biochemical and clinical measurements

Blood samples of all participants were collected after 12–14 h of overnight fasting in a steady-state sitting position between 7:00 and 9.00 a.m. and centrifuged within 30–45 min of collection. All samples were analyzed at the TLGS research laboratory on collection using Selectra 2 auto-analyzer (Vital Scientific, Spankeren, Netherlands). Fasting blood sugar (FBS) was measured using an enzymatic colorimetric method with glucose oxidase. Inter/intra-assay coefficient variations for FBS were both 2.2% for FBS. Triglycerides (TGs) levels were measured using the enzymatic colorimetric method with glycerol phosphate oxidase. Inter- and intra-assay CVs for TGs were 0.6 and 1.6%, respectively.

Blood pressure (BP) was measured twice on the right arm with a minimum interval of 30 s, using a mercury sphygmomanometer and Korotkoff sound technique, with an accuracy of 2 mm Hg, after resting for at least 15-min sitting on a chair. The average of the two measurements was considered the subject’s BP; systolic BP (SBP) with the first sound to be heard and diastolic BP (DBP) with the disappearance of the sound was recorded.

### Dietary assessment

Dietary data were assessed using a valid and reliable semi-quantitative 168-item food frequency questionnaire (24). During a face-to-face interview, the frequency of consumption for each food item during the past year on a daily, weekly, or monthly basis was collected by trained and skilled dieticians. The food items were chosen according to the most frequently consumed items in Iran’s national food consumption survey. Portion sizes of consumed foods reported in domestic measures were then transformed to gram scale. The United States Department of Agriculture (USDA) food composition table (FCT) is used to compute energy and nutrients content (25). The Iranian FCT (26) was used for local food items unavailable in USDA FCT.

For each carbohydrate-containing food, GI is described as the area under the blood glucose response curve over 2 h after eating the food relative to that after consuming the equivalent amount of carbohydrate as glucose. The international table of GI and list of the GI of Iranian foods (27, 28) were used to obtain the GI value of each food item. The total dietary GI was determined as the following:


DietaryGI=[(carbohydratecontentofeachfooditem)

×(number⁢of⁢servings/d)

×(GI)]/totaldailycarbohydrateintake.

### Exposure definition

#### Carbohydrate quality index

The CQI (20) was defined by summing the following four criteria: dietary fiber intake (g/day), GI, whole grains: total grains ratio, and SCHO/TCHO. We considered only the amount of CHO from each food for the determination of dietary SCHO or dietary TCHO. Total grains are calculated by summing the intakes of whole grains, refined grains, and their products. Participants were categorized into quintiles based on intake of each component and were given a value (ranging from 1 to 5) according to each quintile. For fiber, whole grains: total grains ratio, and SCHO/TCHO, subjects in the lowest quintile received 1 point, and those in the highest one received 5 points. For GI, those in the lowest quintile were assigned 5 points, and those in the highest one were assigned 1 point. Finally, we summed up the scores for all four components to compute the CQI score (ranging from 4 to 20). Each component of the score was also considered separately.

#### Protein score

The protein score (12) was set according to total protein intake (% energy) PAR. The study population was divided into 11 strata according to total protein intake and PAR. Participants in the highest category of these components received 10 points, subjects in the next stratum received 9 points, and so on, down to subjects in the lowest category, who received 0 points. Then the points of both components were summed, and the overall protein diet score ranging from 0 to 20 points was created. Each component of the score was also considered separately.

### Physical activity assessment

Physical activity was assessed using the Modifiable Activity Questionnaire (MAQ), which has previously been updated and validated among Iranians (29). Participants were asked to report and identify the frequency and time spent on activities of light, moderate, hard, and very hard intensity during the past 12 months, according to a list of common activities of daily life; physical activity levels were reported as metabolic equivalent hours per week (MET-h/week).

### Outcome definition

The Chronic Kidney Disease Epidemiology Collaboration (CKD-EPI) equation formula was used to express estimated GFR (eGFR) in ml/min/1.73 m^2^ of body surface area (30). The abbreviated CKD-EPI equation is as follows: GFR = 141 × min (serum creatinine/0.7 for females and 0.9 for males, 1)^–1.209^ × 0.993*^Age^* × 1.018 [if female]. CKD was defined as having eGFR of <60 ml/min/1.73 m^2^, and participants with eGFR of ≥60 ml/min/1.73 m^2^ were considered non-CKD.

### Statistical analyses

Statistical analysis was conducted using the SPSS software (Statistical Package for the Social Sciences, version 15.0, SPSS Inc., Chicago, IL, USA). Study populations were categorized into tertiles of protein score, CQI, and their components. Baseline characteristics of participants are expressed as mean ± SD or median (25–75 interquartile range) for continuous variables and percentages for categorical variables across tertiles of protein score and CQI. To test the trend of continuous and dichotomous variables across tertiles of protein score and CQI, linear regression and Chi-square test were performed, respectively. Individuals’ person-time (person-year) and duration of follow-up (in the year) were calculated from baseline to the time at which an event (definitive diagnosis of CKD by a physician based on the above-mentioned criteria) occurred for the first time (event date), or the last date of follow-up examination, whichever occurred first. The event date of occurrence of the CKD was determined as mid-time between the date of the follow-up visit at which the CKD was detected for the first time and the most recent follow-up visit preceding the diagnosis. Cox proportional hazard regression was used to estimate the hazard ratio (HR) and 95% confidence intervals (CIs) of CKD across tertiles of protein score, CQI, and their components according to three models, including model 1 (adjusted for age and sex), model 2 (adjusted for model 1 and BMI, smoking, physical activity, education level, and energy intake) and model (adjusted for model 2 and baseline GFR, SBP, FBS, TGs, and sodium).

We also conducted sensitivity analyses among the three populations without diabetic, hypertensive, and MetS patients to assess the robustness of the study findings. All *P*-values were based on two-sided tests, and *P*-values < 0.05 were considered significant.

## Results

The mean ± standard deviation (SD) of age and BMI of participants (45.7% male) were 37.9 ± 12.8 years and 26.8 ± 4.7 kg/m^2^, respectively. During the 7.7 ± 2.7 years of follow-up, 1,216 cases (20.1%) of CKD occurred.

General characteristics and dietary intake of the study population based on tertiles of protein score are indicated in Table 1. Across tertiles of protein score, the male percentage, BMI, SBP, FBS, TGs, and dietary intake of carbohydrates, protein, PAR, protein score fiber, whole-grain/total-grain, SCHO/TCHO, and CQI increased, while smokers, GFR, dietary intake of energy, fat, and GI decreased. There was no significant difference in physical activity and education status between tertiles of protein score.

**TABLE 1:** Baseline characteristics of participants according to tertiles (T) of protein score.

Variables	Protein score			
	
	T1 (*n* = 2,561)	T2 (*n* = 1,714)	T3 (*n* = 1,769)	*P*_trend_ *
Age (years)	36.1 ± 12.1	37.8 ± 12.8	40.4 ± 13.3	< 0.001
Male (%)	42.3	45.1	51.2	< 0.001
BMI (kg/m^2^)	26.4 ± 4.7	27.0 ± 4.8	27.1 ± 4.6	< 0.001
Smoking (%)	13.2	12.4	11.2	0.049
Physical activity (MET/h/week)	72.7 ± 60.8	74.7 ± 64.2	75.4 ± 64.0	0.159
Academic education (%)	22.6	26.1	24.6	0.086
GFR (ml/min/1.73 m^2^)	80.5 ± 12.3	79.7 ± 12.3	78.9 ± 12.4	< 0.001
SBP (mm Hg)	109 ± 15.2	111 ± 16.0	113 ± 16.2	< 0.001
FBS (mg/dl)	90.3 ± 18.8	91.7 ± 20.4	94.8 ± 26.2	< 0.001
TGs (mg/dl)	134 ± 82.2	142 ± 90.8	153 ± 94.4	< 0.001
**Dietary intakes**				
Energy (Kcal/day)	2,388 ± 713	2,314 ± 727	2,338 ± 710	0.012
Carbohydrate (% of energy)	55.9 ± 6.7	58.4 ± 6.7	61.5 ± 9.8	< 0.001
Fat (% of energy)	33.4 ± 6.5	29.6 ± 5.7	27.7 ± 21.4	< 0.001
Protein score	7.0 ± 1.9	10.4 ± 0.4	13.9 ± 1.9	< 0.001
Protein (% of energy)	13.1 ± 1.8	14.8 ± 3.2	16.1 ± 9.8	< 0.001
PAR	0.93 ± 0.35	1.34 ± 1.01	1.69 ± 0.92	< 0.001
CQI	10.4 ± 2.8	12.0 ± 2.9	14.2 ± 2.9	< 0.001
Fiber (g/1,000 Kcal)	15.4 ± 5.3	19.0 ± 7.9	23.5 ± 25.2	< 0.001
Glycemic index	60.7 ± 7.5	58.7 ± 8.1	55.0 ± 8.1	< 0.001
Whole-grain/total-grain	0.19 ± 0.14	0.26 ± 0.17	0.39 ± 0.20	< 0.001
SCHO/TCHO	0.97 ± 0.03	0.97 ± 0.02	0.98 ± 0.01	< 0.001

BMI, body mass index; GFR, glomerular filtration rate; SBP, systolic blood pressure; FBS, fasting blood sugar; TGs, triglycerides; PAR, plant-to-animal protein ratio; CQ, carbohydrates quality index; SCHO, solid carbohydrates; TCHO, total carbohydrates (including solid carbohydrates + liquid carbohydrates). Data represented as mean ± SD, or median (IQR 25–75) for continuous variables and percent for categorical variables. *Chi-square and linear regression were used to test the trend of continuous and categorical variables across tertiles of the protein quality (as the median value in each tertile), respectively.

Table 2 presents participants’ demographic characteristics and dietary intakes across tertiles of carbohydrate quality. Across the CQI, BMI, SBP, FBS, TGs, dietary intake of carbohydrate, protein, PAR, protein score, fiber, whole grain/total grain, SCHO/TCHO, and CQI increased, and the percent of male and smokers, GFR, dietary intake of fat, and GI decreased. We observed no significant difference in physical activity, education status, and energy intake between tertiles of CQI.

**TABLE 2:** Baseline characteristics of participants according to tertiles (T) of carbohydrates quality index.

Variables	Carbohydrates quality index			*P*_trend_ *
		
	T1 (*n* = 2,014)	T2 (*n* = 2,014)	T3 (*n* = 2,016)	
Age (years)	34.0 ± 11.6	37.9 ± 12.5	41.5 ± 13.0	< 0.001
Male (%)	51.4	44.1	41.5	< 0.001
BMI (kg/m^2^)	26.0 ± 4.7	26.8 ± 4.6	27.5 ± 4.7	< 0.001
Smoking (%)	16	12	9.2	< 0.001
Physical activity (MET/h/week)	75.3 ± 62.6	72.9 ± 61.9	74.0 ± 63.6	0.517
Academic education (%)	24.9	24.8	22.8	0.115
GFR (ml/min/1.73 m^2^)	81.9 ± 12.2	79.9 ± 12.4	77.6 ± 12.1	< 0.001
SBP (mm Hg)	109 ± 15.0	110 ± 15.8	112 ± 16.3	< 0.001
FBS (mg/dl)	90.1 ± 17.3	91.4 ± 20.6	94.6 ± 26.2	< 0.001
TGs (mg/dl)	136 ± 88.0	141 ± 88.8	149 ± 88.8	< 0.001
**Dietary intakes**				
Energy (Kcal/day)	2,351 ± 740	2,347 ± 719	2,358 ± 691	0.763
Carbohydrate (% of energy)	57.3 ± 6.9	58.0 ± 6.6	59.4 ± 10.1	< 0.001
Fat (% of energy)	31.2 ± 6.8	30.7 ± 6.4	30.1 ± 20.3	0.012
Protein score	8.2 ± 2.8	9.6 ± 2.8	12.0 ± 3.1	< 0.001
Protein (% of energy)	13.7 ± 3.4	14.2 ± 2.6	15.5 ± 9.1	< 0.001
PAR	1.09 ± 0.63	1.23 ± 0.74	1.49 ± 1.02	< 0.001
CQI	8.3 ± 1.4	11.9 ± 0.9	15.7 ± 1.5	< 0.001
Fiber (g/1,000 Kcal)	15.7 ± 6.4	19.0 ± 8.1	21.6 ± 23.6	< 0.001
Glycemic index	64.0 ± 6.5	59.0 ± 7.0	52.3 ± 6.5	< 0.001
Whole-grain/total-grain	0.13 ± 0.09	0.24 ± 0.14	0.43 ± 0.18	< 0.001
SCHO/TCHO	0.96 ± 0.03	0.97 ± 0.02	0.98 ± 0.01	< 0.001

BMI, body mass index; GFR, glomerular filtration rate; SBP, systolic blood pressure; FBS, fasting blood sugar; TGs, triglycerides; PAR, plant-to-animal protein ratio; CQ, carbohydrates quality index; SCHO, solid carbohydrates; TCHO, total carbohydrates (including solid carbohydrates + liquid carbohydrates). Data represented as mean ± SD, or median (IQR 25–75) for continuous variables and percent for categorical variables. *Chi-square and linear regression were used to test the trend of continuous and categorical variables across tertiles of the protein quality (as the median value in each tertile), respectively.

**FIGURE 2:**
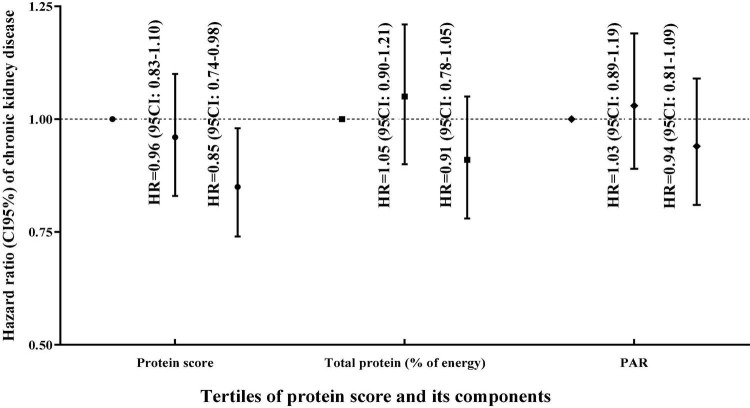
Hazard ratio (95% confidence interval) of chronic kidney disease across tertile protein score and its components based on the multivariable model.

The HR (95% CI) of CKD incident in tertiles of protein score and its components are shown in Table 3. A higher dietary protein score was significantly associated with a lower risk of CKD incident in all Cox regression models. In the final adjusted model for potential confounders, the HR (95% CI) of CKD incident among participants in the highest vs. lowest tertile of protein score was 0.85 (0.74–0.98), *P*_trend_ = 0.033. However, in all adjusted models (Figure 2), total protein was not significantly related to the risk of CKD. The HR (95% CI) of CKD was 0.91 (0.78–1.05), *P*_trend_ = 0.177 for participants in the highest compared with the lowest tertile of total protein in the final adjusted model. Although the PAR was significantly associated with a lower risk of CKD in adjusted models 1 (HR: 0.84; 95% CI: 0.73–0.98), (*P*_trend_ = 0.019), and 2 (HR: 0.85; 95% CI: 0.74–0.99), (*P*_trend_ = 0.030), after adjusting the baseline GFR, SBP, and serum levels of FBS TGs, and sodium intake in the final model, this association become non-significant (HR: 0.94; 95% CI: 0.81–1.090), (*P*_trend_ = 0.439).

**TABLE 3:** Hazard ratio (95% confidence interval) of chronic kidney disease across tertile protein score and its components.

Dietary indices	Protein score and its components			*P*_trend_ *
		
	Tertile 1	Tertile 2	Tertile 3	
**Protein score**				
Median score	7.0	10.0	13.0	
Follow-up period (years)	8.5	8.5	8.6	
Model 1*	1.00 (Ref)	0.96 (0.83–1.11)	0.81 (0.70–0.93)	0.004
Model 2^†^	1.00 (Ref)	0.95 (0.82–1.10)	0.81 (0.70–0.93)	0.005
Model 3^‡^	1.00 (Ref)	0.96 (0.83–1.10)	0.85 (0.74–0.98)	0.033
**Total protein (% of energy)**				
Median score	12.0	14.1	16.5	
Follow-up period	8.4	8.5	8.6	
Model 1*	1.00 (Ref)	1.06 (0.91–1.22)	0.91 (0.78–1.05)	0.156
Model 2^†^	1.00 (Ref)	1.04 (0.90–1.21)	0.89 (0.77–1.03)	0.101
Model 3^‡^	1.00 (Ref)	1.05 (0.90–1.21)	0.91 (0.78–1.05)	0.177
**PAR**				
Median score	0.67	1.07	1.77	
Follow-up period	8.5	8.5	8.6	
Model 1*	1.00 (Ref)	0.96 (0.83–1.11)	0.84 (0.73–0.98)	0.019
Model 2^†^	1.00 (Ref)	0.96 (0.83–1.11)	0.85 (0.74–0.99)	0.030
Model 3^‡^	1.00 (Ref)	1.03 (0.89–1.19)	0.94 (0.81–1.09)	0.439

PAR, plant-to-animal protein ratio. *Model 1: adjusted for age and sex. ^†^Model 2: additionally adjusted for model 1 and body mass index, smoking (yes, no), physical activity, education level (academic, non-academic), and energy intake. ^‡^Model 3: additionally adjusted for model 2 and baseline GFR, systolic blood pressure, fasting blood sugar, triglycerides, and sodium intake (all continues).

**FIGURE 3:**
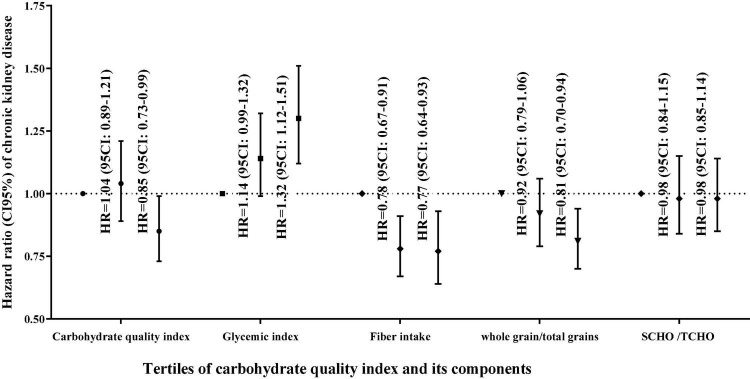
Hazard ratio (95% confidence interval) of chronic kidney disease across carbohydrate quality index score and its components based on the multivariable model.

Table 4 shows the association of carbohydrate score and its components with the incidence of CKD. In all adjusted models, higher carbohydrate score and ratio of whole grain/total grains were significantly related to a lower risk of CKD incident, but higher GI was associated with higher CKD risk. In the final adjusted model for potential confounders, the HR (95% CI) for incidence of CKD among participants who were in the highest compared to lowest tertile of carbohydrate score, the ratio of whole grain/total grains, and GI were 0.85 (0.73–0.99), *P*_trend_ = 0.016 and 0.81 (0.70–0.94), *P*_trend_ = 0.004, and 1.30 (1.12–1.512), *P*_trend_ < 0.001. However, in all adjusted models (Figure 3), SCHO/TCHO showed no significant association with the risk of CKD. The HR (95% CI) of CKD was 0.98 (0.85–1.14), *P*_trend_ = 0.873 for participants in the highest vs. lowest tertile of SCHO/TCHO in the final adjusted model. There was no significant relationship between higher fiber intake and the risk of CKD incident in the age and sex-adjusted model, whereas participants in the highest vs. lowest tertiles of fiber higher significantly associated with a lower risk of CKD in the second [HR (95% CI) = 0.82 (0.68–0.99), *P*_trend_ = 0.063] and third [HR (95% CI) = 0.77 (0.64–0.93), *P*_trend_ = 0.021] adjusted models.

**TABLE 4:** Hazard ratio (95% confidence interval) of chronic kidney disease across carbohydrate quality index score and its components.

Dietary indices	Carbohydrate quality index score and its components			*P*_trend_ *
		
	Tertile 1	Tertile 2	Tertile 3	
**Carbohydrate quality index**				
Median score	8.00	12.00	16.00	
Follow-up period	8.5	8.5	8.6	
Model 1*	1.00 (Ref)	1.06 (0.91–1.24)	0.83 (0.71–0.97)	0.007
Model 2^†^	1.00 (Ref)	1.06 (0.90–1.24)	0.83 (0.71–0.97)	0.006
Model 3^‡^	1.00 (Ref)	1.04 (0.89–1.21)	0.85 (0.73–0.99)	0.016
**Glycemic index**				
Median score	50.7	58.5	66.3	
Follow-up period	8.4	8.6	8.5	
Model 1*	1.00 (Ref)	1.11 (0.96–1.28)	1.19 (1.03–1.38)	0.015
Model 2^†^	1.00 (Ref)	1.13 (0.98–1.30)	1.21 (1.05–1.40)	0.008
Model 3^‡^	1.00 (Ref)	1.14 (0.99–1.32)	1.30 (1.12–1.51)	< 0.001
**Fiber intake**				
Median score	13.0	17.4	23.0	
Follow-up period	8.4	8.6	8.5	
Model 1*	1.00 (Ref)	0.91 (0.79–1.06)	0.93 (0.80–1.07)	0.399
Model 2^†^	1.00 (Ref)	0.85 (0.73–1.00)	0.82 (0.68–0.99)	0.063
Model 3^‡^	1.00 (Ref)	0.78 (0.67–0.91)	0.77 (0.64–0.93)	0.021
**Whole grain/total grains**				
Median score	0.07	0.23	0.46	
Follow-up period	8.4	8.6	8.5	
Model 1*	1.00 (Ref)	0.88 (0.76–1.02)	0.82 (0.71–0.94)	0.010
Model 2^†^	1.00 (Ref)	0.87 (0.75–1.01)	0.82 (0.71–0.94)	0.010
Model 3^‡^	1.00 (Ref)	0.92 (0.79–1.06)	0.81 (0.70–0.94)	0.004
**SCHO/TCHO**				
Median score	0.96	0.98	0.99	
Follow-up period	8.4	8.6	8.5	
Model 1*	1.00 (Ref)	0.98 (0.84–1.15)	0.90 (0.78–1.05)	0.220
Model 2^†^	1.00 (Ref)	0.99 (0.85–1.16)	0.91 (0.78–1.06)	0.269
Model 3^‡^	1.00 (Ref)	0.98 (0.84–1.15)	0.98 (0.85–1.14)	0.873

SCHO/TCHO, solid carbohydrates/total carbohydrates ratio. *Model 1: adjusted for age and sex. ^†^Model 2: additionally adjusted for model 1 and body mass index, smoking (yes, no), physical activity, education level (academic, non-academic), and energy intake. ^‡^Model 3: additionally adjusted for model 2 and baseline GFR, systolic blood pressure, fasting blood sugar, triglycerides, and sodium (all continues).

Table 5 shows the results of sensitivity analyses among three populations of the whole study population for assessing the robustness of findings of protein score and CQI relationship and CKD incidence. By excluding the diabetic patients in baseline, The HR (95% CI) of CKD incidence among participants in the highest vs. lowest tertile of CQI, GI, fiber intake, and whole grain/total grains was 0.84 (0.71–0.99), 1.26 (1.08–1.48), 0.79 (0.64–0.97), and 0.81 (0.69–0.94), respectively. When hypertensive patients were excluded, still CQI (HR: 0.80; 95% CI: 0.67–0.96) and whole grain/total grains (HR: 0.81; 95% CI: 0.69–0.96) were associated with a lower risk of CKD, whereas higher GI score (HR: 1.32; 95% CI: 1.11–1.57) was related with a higher risk of CKD. Also, When patients with MetS were excluded, still CQI (HR: 0.78; 95% CI: 0.64–0.95) and whole grain/total grains (HR: 0.79; 95% CI: 0.65–0.95) were related to lower risk of incidence of CKD; however, higher GI score (HR: 1.39; 95% CI: 1.15–1.68) was associated with higher CKD risk.

**TABLE 5:** The association of protein score, carbohydrate quality index score, and their components with risk of chronic kidney disease.

	Hazard ratios (95% confidence intervals)			*P*_trend_ *
		
	Tertile 1	Tertile 2	Tertile 3	
**Diabetic patients excluded (*N* = 5,321)**				
Protein score	1.00 (Ref)	0.92 (0.79–1.08)	0.87 (0.75–1.02)	0.103
Total protein (% of energy)	1.00 (Ref)	1.06 (0.90–1.20)	0.92 (0.78–1.08)	0.277
PAR	1.00 (Ref)	1.03 (0.88–1.21)	0.95 (0.81–1.11)	0.562
Carbohydrate quality index	1.00 (Ref)	1.03 (0.87–1.22)	0.84 (0.71–0.99)	0.021
Glycemic index	1.00 (Ref)	1.12 (0.96–1.31)	1.26 (1.08–1.48)	0.003
Fiber intake	1.00 (Ref)	0.81 (0.68–0.95)	0.79 (0.64–0.97)	0.045
Whole grain/total grains	1.00 (Ref)	0.91 (0.77–1.07)	0.81 (0.69–0.94)	0.009
SCHO/TCHO	1.00 (Ref)	0.97 (0.82–1.15)	0.96 (0.81–1.13)	0.639
**Hypertensive patients excluded (*N* = 5,153)**				
Protein score	1.00 (Ref)	0.99 (0.84–1.17)	0.91 (0.77–1.08)	0.320
Total protein (% of energy)	1.00 (Ref)	1.09 (0.92–1.30)	0.93 (0.78–1.11)	0.378
PAR	1.00 (Ref)	1.04 (0.88–1.23)	0.93 (0.78–1.11)	0.457
Carbohydrate quality index	1.00 (Ref)	0.98 (0.82–1.18)	0.80 (0.67–0.96)	0.009
Glycemic index	1.00 (Ref)	1.81 (0.99–1.39)	1.32 (1.11–1.57)	0.001
Fiber intake	1.00 (Ref)	0.81 (0.67–0.97)	0.82 (0.66–1.03)	0.154
Whole grain/total grains	1.00 (Ref)	0.88 (0.74–1.04)	0.81 (0.69–0.96)	0.019
SCHO/TCHO	1.00 (Ref)	0.99 (0.82–1.19)	0.91 (0.76–1.09)	0.342
**Metabolic syndrome patients excluded (*N* = 4,348)**				
Protein score	1.00 (Ref)	0.87 (0.72–1.05)	0.84 (0.70–1.02)	0.077
Total protein (% of energy)	1.00 (Ref)	1.06 (0.88–1.28)	0.90 (0.74–1.09)	0.255
PAR	1.00 (Ref)	1.07 (0.89–1.29)	0.89 (0.73–1.08)	0.265
Carbohydrate quality index	1.00 (Ref)	0.90 (0.73–1.10)	0.78 (0.64–0.95)	0.015
Glycemic index	1.00 (Ref)	1.07 (0.89–1.29)	1.39 (1.15–1.68)	0.001
Fiber intake	1.00 (Ref)	0.77 (0.63–0.94)	0.76 (0.59–0.98)	0.063
Whole grain/total grains	1.00 (Ref)	0.82 (0.68–1.00)	0.79 (0.65–0.95)	0.021
SCHO/TCHO	1.00 (Ref)	0.91 (0.74–1.12)	0.88 (0.72–1.08)	0.236

*Model 1: adjusted for age and sex. ^†^Model 2: additionally adjusted for model 1 and body mass index, smoking (yes, no), physical activity, education level (academic, non-academic), and energy intake. ^‡^Model 3: additionally adjusted for model 2 and baseline GFR, systolic blood pressure, fasting blood sugar, triglycerides, and sodium (all continues).

## Discussion

In this prospective cohort study, after 7.7 years of follow-up, we observed that a diet with a high protein score and high quality of carbohydrates was significantly related to decreased risk of CKD, independent of important confounders, including age, sex, smoking, total energy intake, BMI, physical activity, baseline eGFR, SBP, serum FBS, and TGs. Also, the higher ratio of whole grain/total grains and fiber intake was significantly associated with decreased risk of CKD. However, the higher dietary GI is related to higher CKD incidents. Furthermore, there is no significant association between total protein, PAR, and SCHO/TCHO and CKD risk.

Although so far, no study has yet assessed the association of protein score and CQI with the risk of CKD, our results are comparable with the findings of several previous studies that have focused on the possible relationship between quality and quantity of protein with eGFR level or renal dysfunction (3, 11–13). Yuzbashian et al. reported that a higher intake of plant protein and lower animal protein intake was positively related to eGFR level and reduced the risk of CKD (11). Also, Møller et al. reported a positive association between higher protein scores and increased GFR levels (12). The Alvirdizadeh et al. study did not show any significant relationship between total and animal protein and the risk of CKD, however, they reported that higher plant protein consumption may be associated with a reduced risk of CKD incident (3). Furthermore, results of a large cohort study on the USA population indicate that although higher intakes of plant and animal protein are independently associated with cardiometabolic indices, no remarkable association was observed between protein intakes and the risk of renal dysfunction (13). The differences in some of the results of previous studies and our study on the role of quality and quantity of dietary protein in the risk of kidney dysfunction can be explained by various reasons; the difference in protein content of dietary patterns and its food sources and the method of preparing and cooking foods containing high protein in various societies can be act as a major source of existing controversies. Differences in general individual characteristics such as gender, age, race, and basic metabolic conditions of the population at the beginning of the study and differences in study design and adjustment of confounding factors were the other reasons that may explain the possible controversies in the findings of previous investigations.

Our findings support the hypothesis that adherence to a plant-based protein diet, along with lower intakes of animal protein, can play a crucial role in preventing the decrease in GFR and reducing the risk of developing kidney damage. The Lin et al. study reported that higher consumption of red meat (two or more servings per week), which is rich in animal protein and fat, may be associated with renal dysfunction (31).

Indeed, high consumption of red and processed meat as a food item rich in saturated fatty acids and sodium is recognized as part of an inappropriate dietary pattern and unhealthy lifestyle (11) that can be associated with an increased risk of CKD (32). The Haring et al. study (33) suggested that specific dietary protein sources may play a different role in the progression of CKD; they revealed that dietary pattern rich in red and processed meat was adversely related to CKD risk, but higher intakes of plant-based proteins from food sources such as nuts and legumes play a protective role against the progression of CKD (33). Some reasons and explanations may support our results that suggest the possible relationship between plant protein and animal protein with renal function; higher intakes of protein from various plant food sources, including gluten (34) and soy protein (35), may decrease the levels of serum TGs, total cholesterol, and reduce oxidized low-density lipoprotein-cholesterol and uric acid, therefore, plant protein can be effective in decreasing oxidized-lipoprotein–induced glomerular damage and reducing the development of CKD by reducing these lipids concentration (36). The different amino acid composition of plant protein compared to animal protein (higher proportions of glutamic acid, phenylalanine, proline, cysteine, and serine) can also play an effective role in kidney function and its related risk factors, such as BP (37). A diet with high plant-based protein is also rich in antioxidant nutrients such as vitamin C, potassium, calcium, and magnesium, and phytochemicals that can be linked to lower dietary acid load and improved renal function (38, 39).

Although the association of CQI (focusing on quantity and quality of CHOs) with eGFR level or renal dysfunction has yet not been assessed, our findings are somewhat comparable with limited studies with controversial results that previously have focused on the possible role of CHO with risk of chronic disease (14, 21). Zazpe et al. suggested that a better quality of dietary CHO (higher score of CQI) is inversely associated with the risk of CVD incidence (14). Also, Kim et al. have reported that a higher CQI is negatively related to the risk of obesity and HTN, however, it was not significantly associated with diabetes and MetS (21). Furthermore, our results are in agreement with previous results among the Iranian population that revealed an inverse relationship between higher dietary fiber and the risk of CKD (11, 40).

A high CQI diet is characterized by a high intake of whole grains and fiber, a low GI, and a lower intake of simple carbohydrates, so it may reduce the risk of CKD based on its different characteristics. It was previously reported that higher adherence to a diet with high GI is associated with the likelihood of having eGFR <60 ml/min/1.73 m^2^ by 55%, but the higher intake of dietary fiber was related to a 50% lower CKD risk (41). Also, increased intakes of simple sugar from sugar-sweetened beverages may increase the risk of CKD incidents (42, 43).

Dietary fiber is negatively associated with hyperuricemia and uric acid concentration, which are important risk factors for the progression of kidney disease (11, 44). Also, there is an inverse association between dietary fiber and the homeostasis model assessment-estimated insulin resistance (HOMA-IR); previous evidence has shown the possible role of HOMA-IR in increasing the risk for CKD by 70% (45). Furthermore, dietary fiber can reduce the progression of kidney disease in susceptible and high-risk individuals by improving glycemic control, increasing insulin sensitivity, decreasing oxidative stress, and reducing the GI of the diet (40, 46, 47). Finally, high intakes of fiber from its rich resources, including vegetables, whole grains, and legumes, may reduce the risk of CKD incidents by attenuating known CKD risk factors, including HTN, obesity, diabetes, hyperlipidemia, and reducing systemic inflammation (25, 40, 48).

The important strength of the current study should be noted; this is the first study that examined the association of dietary protein score and CQI with the risk of CKD using a large sample size, population-based prospective cohort study. Dietary data were collected using a valid and reliable FFQ, which reduces the risk of measurement bias in dietary data.

However, our study had some limitations that need to be mentioned. As in most epidemiological studies, we measured serum creatinine once in the study participants to determine the possibility of CKD incident, while measuring creatinine three times could have enhanced the accuracy of detecting CKD. The possibility of measurement error is somewhat unavoidable despite using the validated FFQ in estimating participants’ dietary intakes. Although we have controlled the possible confounding effects of several variables in the final analysis of the current study, residual confounding due to unknown or unmeasured variables, such as albuminuria/proteinuria and renal protecting drugs [such as angiotensin-converting enzyme inhibitors (ACEIs)/angiotensin receptor blockers (ARBs) or use of SGLT2 inhibitors] cannot be excluded. In conclusion, our results revealed that greater adherence to a dietary pattern with a high protein score and high quality of carbohydrates is characterized by higher intakes of plant proteins, whole grains, and fibers and lower intakes of animal proteins, high GI carbohydrates, and simple sugars, may be associated with reduced risk of CKD incident. Further studies are recommended in other populations to clarify the possible role of carbohydrate and protein quality and quantity in predicting the risk of CKD.

## Data availability statement

The raw data supporting the conclusions of this article will be made available by the authors, without undue reservation.

## Ethics statement

Written informed consent was obtained from the individual(s) for the publication of any potentially identifiable images or data included in this article.

## Author contributions

FT and MV conceptualized and designed the study and analyzed and interpreted the data. FT, HF, and HA drafted the initial manuscript. HF and MJ contributed to the revision of the manuscript. MV, PM, and FA supervised the project. All authors have read and approved the final version of the manuscript.
